# Dynamic Correlation Analysis Method of Air Pollutants in Spatio-Temporal Analysis

**DOI:** 10.3390/ijerph17010360

**Published:** 2020-01-05

**Authors:** Yu-ting Bai, Xue-bo Jin, Xiao-yi Wang, Xiao-kai Wang, Ji-ping Xu

**Affiliations:** 1 School of Computer and Information Engineering, Beijing Technology and Business University, Beijing 100048, China; baiyuting@btbu.edu.cn (Y.-t.B.); xujiping@139.com (J.-p.X.); 2 Beijing Key Laboratory of Big Data Technology for Food Safety, Beijing Technology and Business University, Beijing 100048, China; 3 College of Physics and Electronic Engineering, Shanxi University, Taiyuan 030006, China; wxk2000@263.net

**Keywords:** correlation degree, spatio-temporal analysis, air pollution management, pollutant source tracing

## Abstract

Pollutant analysis and pollution source tracing are critical issues in air quality management, in which correlation analysis is important for pollutant relation modeling. A dynamic correlation analysis method was proposed to meet the real-time requirement in atmospheric management. Firstly, the spatio-temporal analysis framework was designed, in which the process of data monitoring, correlation calculation, and result presentation were defined. Secondly, the core correlation calculation method was improved with an adaptive data truncation and grey relational analysis. Thirdly, based on the general framework and correlation calculation, the whole algorithm was proposed for various analysis tasks in time and space, providing the data basis for ranking and decision on pollutant effects. Finally, experiments were conducted with the practical data monitored in an industrial park of Hebei Province, China. The different pollutants in multiple monitoring stations were analyzed crosswise. The dynamic features of the results were obtained to present the variational correlation degrees from the proposed and contrast methods. The results proved that the proposed dynamic correlation analysis could quickly acquire atmospheric pollution information. Moreover, it can help to deduce the influence relation of pollutants in multiple locations.

## 1. Introduction

In the rapid expansion of society and economy, pollutants and sources are emerging as threats to indoor and outdoor air quality, although various measures have been conducted to control pollution. In practice, many information systems are established to monitor pollutant discharge. The systems usually provide the functions of real-time monitoring and trend prediction. The functions provide only the basic information for the administrator and decision maker. Moreover, the influence relation is important for the management of environment and public health [1,2]. There is an urgent demand to explore the influence relation of pollutants and the potential pollution sources. The paper focused on the analysis method of pollutants and sources, which can provide a solution to the emerging issues in air quality management.

The issue of pollutant relation and source tracing belongs to the spatial and temporal analysis of atmospheric variables [3,4]. For studying the issue, some explored fluid mechanics and probability models, such as Gaussian plume model [5], Gaussian puff model [6], state-space model [7] and hidden Markov model [8]. The models simulate the gas diffusion process from the source to the surrounding area. The category of the models is built on mechanism analysis, which relies heavily on the professional knowledge of environmental sciences and physics. Besides, for the demand of source tracing, the models are difficult to apply reversely, that is, to find out the pollution source with the gas distribution. The other category of analysis methods is the data-driven solution. The implicit information is extracted from data with statistical and information processing methods, such as the spatial–temporal statistics [9,10,11], functional data analysis [12,13], and correlation analysis [14,15,16]. The spatial–temporal statistics focus on the statistical parameters from the historical data. The functional data analysis can build the regressive model with the data feature. The correlation analysis focuses on the numerical relationship of variables with an intuitional and lucid correlation degree. The methods above rely on a certain amount of data, and they output a general condition for a period. They are short of the timeliness and dynamic features.

Different deficiencies exist in the methods above, which will be introduced in detail in the Section of Related Work. For atmospheric environment management, there are some practical problems. Firstly, air pollutants change obviously in a season and even in a day. Secondly, the pollutant diffusion is impacted by production activities in industrial parks. The different factories can lead to diversiform gas diffusion. Thirdly, there is the cross-impact of multiple variables on a point, as well as multiple positions. The complicated interaction effect is a severe problem in practical analysis. In brief, there is a gap between practical demand and the existing methods. The correlation must be analyzed dynamically in real-time. Besides, the spatial correlation should be conducted to excavate the pollution source information intuitively and rapidly.

For the problems above, a dynamic spatio-temporal correlation analysis method is proposed in a data-driven thought. The method in this paper emphasizes the correlation degree of pollutant variables and positions, of which the process runs dynamically, and the results are direct for influence relation and source tracing. The method is designed considering the inference of multiple positions in the spatial dimension, and the dynamic real-time calculation in the temporal dimension. The case experiment is carried out with the monitoring data of an industrial park in Hebei Province, China.

The rest of this paper is organized as follows. Section 2 introduces the related work, including the spatial distribution model and correlation analysis method. In Section 3, the main spatio-temporal framework and method are proposed. Experiments are conducted in Section 4, and the results are discussed in Section 5. Finally, the study of the paper is concluded in Section 6.

## 2. Related Work

As mentioned in the Introduction, the main tools to analyze air pollutants in the spatial and temporal dimensions include the gas diffusion model, spatial–temporal statistics, functional data analysis, and correlation analysis method. The basic principle and related studies are presented in this section. They are also analyzed under the management demand of an industrial atmospheric environment.

### 2.1. Spatio-Temporal Analysis Method

#### 2.1.1. Gas Spatial Diffusion Model

The spatial distribution is a fundamental feature of the atmospheric elements. It plays a vital role in the analysis of pollutant diffusion and surrounding influence. The classical models have been built for the gas diffusion analysis, in which the Gaussian plume model [5] and the Gaussian puff model [6] have been the representatives, based on the probability model. The probability model makes posterior probability statistics of gas diffusion at a specific time point through prior probability and judges the diffusion parameters with the probability value. Many researchers use the Gaussian model to calculate the concentration distribution of leakage media under different conditions, as well as the variation rule in the time dimension.

In the study and application of the Gaussian model [17,18,19], some focus is done on the issue of gas diffusion with the known emission source. The default coordinate system is set up taking the emission source as the origin, and the wind direction, and its vertical relations as axes. In the Gaussian model with established parameters, only the position information of three directions and emission time are needed to calculate the gas concentration at the specified position. Besides, others focus on the issue of gas coverage. In the case of specified parameters (standard difference of source strength, etc.) and gas concentration, the approximate gas coverage can be found based on the model.

The leading role of the Gaussian model is the forward analysis, in which the pollutant diffusion and distribution can be obtained based on the source information. However, in demand for pollution source tracing, the back-forward inference is needed to find out the source strength based on the gas distribution. In the back-forward case, the Gaussian model is difficult to reverse because of the hypothetical excess parameters. The reversed model will output different inference results of the source when some of the parameters are inaccurate. Hence, there is a distinct shortage in the diffusion model for the inference of variable influence and source tracing.

#### 2.1.2. Spatial–Temporal Statistics and Functional Data Analysis

Spatial and temporal analysis has drawn attention based on various geographic information systems, including atmospheric monitoring. The classical methods include spatial–temporal statistics and functional data analysis. The spatial–temporal statistics [9,10,11] mainly analyze the mutual structure of spatial distribution and the feature of time series. The spatial distribution pattern is estimated by the first-order (large scale samples) structure and the second-order (small scale or local samples) structure, and the non-sample spatial region is predicted or interpolated by the estimated results. The functional data analysis [12,13] mainly transforms the original discrete data into a functional form, so as to explore the correlation between the data through the analysis of function.

Scholars have applied the spatial–temporal statistics and functional data analysis methods to environmental issues. In the method studies [20,21,22], the statistics parameters are obtained and converted to form functions. The functions can fit the data trends with the least-squares, variance analysis, maximum likelihood estimation, etc. Based on the functions, the data can be analyzed in the mapping relation from the functions.

For the spatial–temporal statistics and functional data analysis methods, there are some difficulties in the application for the real-time analysis demand in our problem. Firstly, the methods mainly realize the analysis during a period. The results are the description and representation of past conditions. It still needs the exploration of the real-time conduction for the methods. Secondly, a fundamental condition of the methods is sufficient data of many points over a long period. The statistics results may be unauthentic if the available samples are not enough. Thirdly, the accurate regression of a function is difficult because of the complex nonlinearity and being nonstationary. The analysis results are mainly impacted by the fitting level of the function based on the data. Hence, the applications of the statistics and functional methods become difficult for various concrete problems in dynamic demand.

### 2.2. Correlation Analysis Method

Correlation analysis of atmospheric pollutants is the simple and effective access to determine the influencing factors and trace the pollution source. In a literature review, the mainstream of correlation analysis methods includes partial correlation [14,23], principal component [15,24], and grey correlation analysis [16,25], which have been applied widely in different fields.

The partial correlation analysis method focuses on the issue of more than three variables. It analyzes the correlation relationship between two variables, independently, without the third one. In partial correlation, the correlation coefficient R or R^2^ is set as the criterion for the correlation degree. Li et al. [23] applied partial correlation analysis to the impact of market elements on the domestic stock market. Porth et al. [26] studied the nutrient resource allocation between plant growth and recuperation based on the partial correlation of gene expressions. Olszewski et al. [27] analyzed the longitudinal correlation between two particles in heavy-ion collisions and extracted the relationship between partial covariance and conditional covariance. It proved the feasibility of the statistical method in the physics field.

Principal component analysis aims at obtaining an independent comprehensive index, namely principal component, by synthesizing a variety of indicators. The principal component index is expected to map almost all the information on the initial data. Calce et al. [28] applied principal component analysis to the standard evaluation of the osteoarthritis. Lionnie et al. [29] established a biometric recognition pattern system, in which principal component analysis extracts features in the mathematical and statistical solution. The cross-validation proved the validity of the fusion method. Cai et al. [30] proposed a detection and location method for disturbances in the power system, in which principal component analysis was fused with k-nearest neighbor analysis.

The grey system theory has been studied widely in various fields. Moreover, the grey relational analysis method is broadly used in the assessment system. Grey relational analysis refers to the quantitative description and comparison of the development and change trend of a system. It determines the closeness by judging the geometric shape similarity of the reference and several comparative data. Fu et al. [31] studied the relationship between the air quality indexes of Beijing and its surrounding region with the grey convex relation model. Cao et al. [32] tried to determine the main influence factors of the atmospheric corrosion of Q235 carbon steel with a grey relational analysis method. Hashemi et al. [33] built a comprehensive green supplier selection model, in which the analysis network process was used to deal with the interdependencies between the criteria, based on the improvement of traditional grey relational analysis. Malekpoor et al. [34] applied grey relational analysis to the sustainable electricity generation planning, in which the evaluation and rank of systems were determined with grey interval values.

It can be found that correlation analysis methods perform differently in concrete applications. An appropriate method should be selected with the specific demand. The grey correlation analysis method has a simple and reliable structure with an appropriate calculation scale. Moreover, there is not an excessive requirement for the sample size. It is more suitable for the demand of real-time and fast analysis. Besides, most of the studies use the methods in a static view, in which a constant correlation number is obtained based on a period of historical data. It is a practical demand to analyze the real-time correlation in different time points. Then, the correlation analysis method should be improved in the dynamic view along time.

## 3. Dynamic Spatio-Temporal Correlation Analysis Method

There are some practical demands for air quality management. Firstly, it is expected to explore and trace the pollution source region, except for the existing real-time monitoring and future prediction. Secondly, the data-driven correlation analysis method can help inferencing the influence variables and possible source region, based on the review of related work. Thirdly, it is needed to obtain the analysis result in time, of which multiple dimensions should be covered, including the pollutant variables and locations. Therefore, the dynamic spatio-temporal correlation analysis method is designed. The general framework and basic dynamic correlation method will be presented firstly, and Then, the spatio-temporal correlation analysis algorithm will be concluded finally.

### 3.1. Spatio-Temporal Correlation Analysis Framework

**Figure 1:**
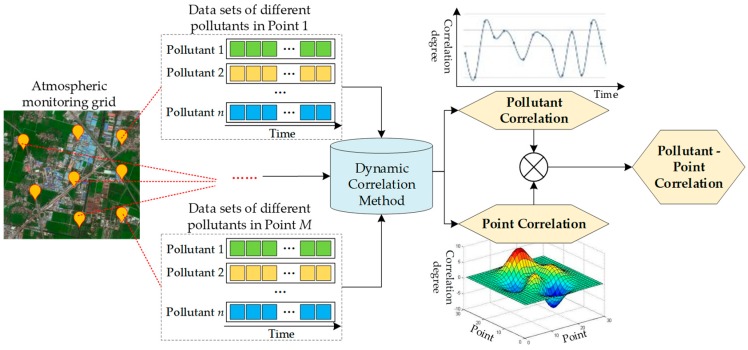
Framework of spatio-temporal correlation analysis on atmospheric pollutants.

Based on the demand analysis of the industrial atmospheric management, the correlation analysis should meet three aspects of needs: (1) the interaction of multiple pollutant variables should be explored, (2) the influence of different locations should be analyzed, and (3) the analysis should be conducted in the real-time based on the monitoring system. Then, a comprehensive correlation analysis framework is designed, as shown in Figure 1. 

The framework in Figure 1 mainly consists of three parts, namely, the data source, core analysis method, and result presentation.

For the data source, the atmospheric monitoring system is set as the infrastructure. Taking the air monitoring grid in China as an example, monitoring stations have been established with a grid layout, in which the equipment is placed at the intersection of the rectangular mesh. The monitoring grid is expected to increase the measurement coverage, and each station can reflect the circumjacent air conditions. The monitoring stations provide data in the framework, and the data consist of multiple pollutant variables with a certain frequency. 

For the core analysis method, a dynamic correlation method is studied in this paper, which is introduced in Section 3.2. The method can output the correlation between the pollutant variables, as well as the correlation between monitoring points.

For the result presentation, various forms can be selected, referring to the data types. The pollutant variable correlation is the time series, which can be shown in the curve graph. The correlation of points has the two-dimensional cross-relation with time features. Moreover, the two types of pollutant and point correlations can be integrated, for example, pollutant variable *A* in point 1 can be analyzed with variable *B* in point 2. Then, the integration result can be queried in an appropriate form.

### 3.2. Dynamic Correlation Calculation

In the spatio-temporal correlation analysis framework, the vital component is the dynamic correlation analysis method. The concrete applications are conducted based on the correlation analysis. For the need of dynamic calculation, the method is studied with information entropy and grey relational analysis.

#### 3.2.1. Adaptive Sliding Window with Information Entropy

In the traditional correlation analysis, the result is static based on all historical data. In the dynamic method, the correlation should be calculated in time with a small time interval. The calculation cannot cover all historical data repeatedly, considering the computing load and speed. Moreover, a sliding window is a useful tool to reduce the calculated amount. However, a fixed-length window may lose efficacy. The data feature may be lost if the window is short, while the computing load may increase if the window is long. Then, information entropy is introduced to improve the sliding window in the adaptive view.

Information entropy can extract data variation characteristics quantitatively and effectively. The change of time-series data can be mapped to a scalar of data fluctuation based on information entropy. Then, a rational threshold can be set to distinguish the data fluctuation range, and it can guide the sliding window length in the correlation analysis.

In the concrete design, the sliding window length should be adjusted according to the time-series features. When the near-term data change smoothly, the sliding window should be lengthened to expand the data range and cover more data characteristics. When the data fluctuate severely, the interception window size should be shortened, the correlation analysis range will be reduced, and the identification of instantaneous regional characteristics will be improved. Meanwhile, the adjustment can improve the calculation efficiency, avoiding redundant computing. In the idea of window adjustment, an adaptive sliding window determination method is proposed based on information entropy [35].

(1) The default window length $L_{0}$ is given firstly, and minimum of $L_{0}$ should be 10, and its maximum should be less than ten percent of the total data number. At each time point, the previous $L_{0}$ of values are used to measure the time series variation. The mean value of the segment is calculated:

(1) $$m=\frac{\sum_{i=1}^{L_{0}}d_{i}}{L_{0}}$$

where i is the time point, m is the mean value of the data segment, $d_{i}$ is the *i*-th value in the data segment.

(2) The variation of the time series is measured with the definition of data fluctuation scalar $z_{i}$:

(2) $$z_{i}=\frac{m}{z_{i}-z_{i-1}}$$

(3) The data fluctuation scalar is converted into a probability measure $p_{i}$, which reflects the change degree of a single point relative to the change degree of whole intercept data segment. And it is converted in the percentage form:

(3) $$p_{i}=\frac{z_{i}}{\sum_{i=1}^{L_{0}-1}z_{i}}$$

(4) The information entropy is applied to transform the probability measure to the data fluctuation characteristic. Concretely, the changes of each point data are transformed to the probability, and information entropy is calculated with change characteristics carried in the intercept data. The information entropy H is calculated as following:

(4) $$H=-\sum_{i=1}^{L_{0}-1}p_{i}\times \log_{2}p_{i}$$

(5) The adjustment proportion of sliding window length is defined as

(5) $$s=\frac{H}{H_{0}}$$

where $H_{0}=\log_{2}L_{0}$ is the maximum information entropy value in the current data segment, and the new window length L is defined as

(6) $$\left\{ \begin{array}{l} L=L_{0},s_{\min}<s<s_{\max}\\ L=\frac{L_{0}}{s},s_{\max}<s\\ L=s\times L_{0},s<s_{\min} \end{array}\right.$$

where $s_{\min}$ and $s_{\max}$ are the stability threshold, and $s_{\min}=\min\{p_{i}\}$, and $s_{\max}=\max\{p_{i}\}$.

#### 3.2.2. Grey Relational Analysis

As introduced in the related work, the grey relational analysis, which is based on grey theory, seeks and defines the quantitative relationship between the factors of a system. It is one of the few methods which can reflect the geometric relationship between the data intuitively. The process of grey relational analysis [16] is introduced briefly here.

(1) Define the object variable y and its potential associated variables $x_{k}$, k is the serial number of associated variables, and 1≤k≤n. The time series values in y and $x_{k}$ are denoted as y(i) and $x_{k}(i)$.

(2) The original data of object variable and associated variables should be normalized to remove the effect of different measurement units.

(3) The object variable y(i) is set as the reference sequence, and a comparison matrix is built by conducting subtraction operation on the reference sequence and the associated variable sequence $x_{k}(i)$.

(4) Calculate the maximum difference between the two levels in the matrix $\underset{k}{\max}\;\underset{i}{\max}|y(i)-x_{k}(i)|$ and the minimum difference $\underset{k}{\min}\;\underset{i}{\min}|y(i)-x_{k}(i)|$.

(5) The item value of each variable corresponding to the reference sequence is obtained, and the mean value of the correlation coefficient is calculated. Then, the correlation sequence $\xi_{k}(i)$ can be formed, as the following formula:

(7) $$\xi_{k}(i)=\frac{\underset{k}{\min}\;\underset{i}{\min}\left|y(i)-x_{k}(i)\right|+\rho \;\underset{k}{\max}\;\underset{i}{\max}\left|y(i)-x_{k}(i)\right|}{\left|y(i)-x_{k}(i)\right|+\rho \;\underset{k}{\max}\;\underset{i}{\max}\left|y(i)-x_{k}(i)\right|}$$

where ρ is the resolution ratio, 0<ρ<1. The greater the difference between correlation coefficients, the stronger the ability to distinguish, in which the difference is positively related with ρ. ρ can be defined as about 0.5 according to the experience.

(6) According to the correlation sequence in Formula (7), the correlation degree between the object variable and the *k*-th associated variable is calculated:

(8) $$r_{k}=\frac{1}{L}\sum_{i=1}^{L}\xi_{k}(i),i=1,2,\ldots ,L$$

where L is the data size in the sliding window determined with the method in Section 3.2.1.

### 3.3. Dynamic Spatio-Temporal Correlation Algorithm

Based on the correlation analysis framework, two basic tasks should be conducted with the correlation analysis methods, including the correlation of variables in one monitoring point and the correlation of different points. The algorithm is designed in this subsection for the two tasks by organizing the theoretical algorithms in Section 3.2 based on the framework in Section 3.1.

**Figure 2:**
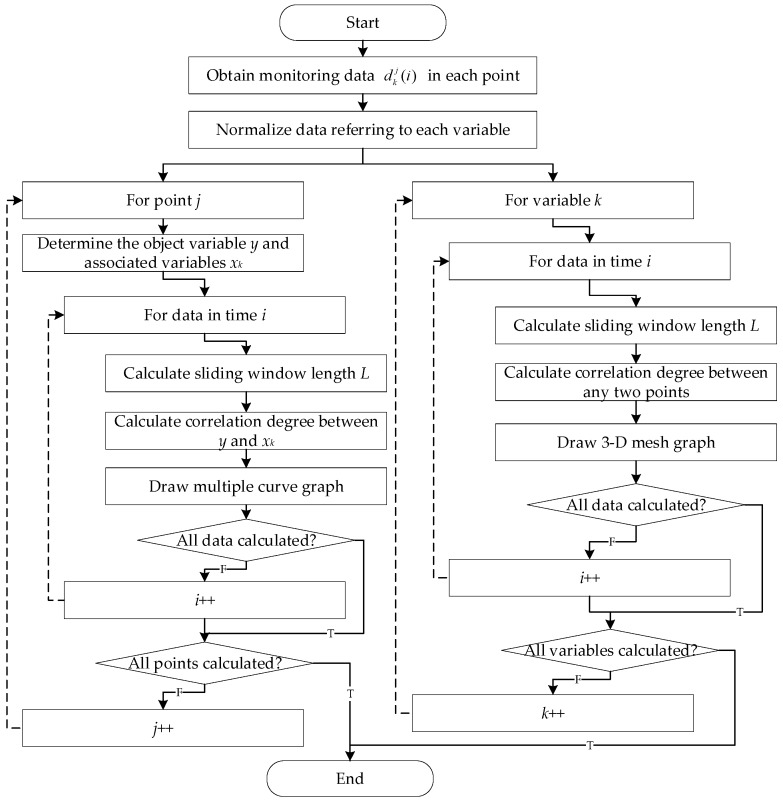
Flow chart of dynamic spatio-temporal correlation algorithm.

The algorithm consists of two parts, one is the single-point pollutant variables correlation, and the other is the multiple points correlation. The flow of the dynamic spatio-temporal correlation algorithm is shown in Figure 2.

For the algorithm shown in Figure 2, the analysis on points and variables are conducted respectively. For the left column, the loop of points is designed to obtain the variable correlation information at each point. For the right column, the loop of variables is for the point correlation information. 

There is the time recurrence in both extrinsic loops to calculate the correlation dynamically. In the time recurrence, the data before the current moment, of which the size is $L_{0}$, are used to determine the sliding window firstly. The window length can be adjusted according to Equations (1)–(6), and the new length is L. Then, the L values before now are used to calculate the correlation degree following the grey relational method in Section 3.2.2. Finally, the results can be presented with different forms which will be shown intuitively in the experiment section.

## 4. Experiment and Result

### 4.1. Dataset and Experiment Setting

**Figure 3:**
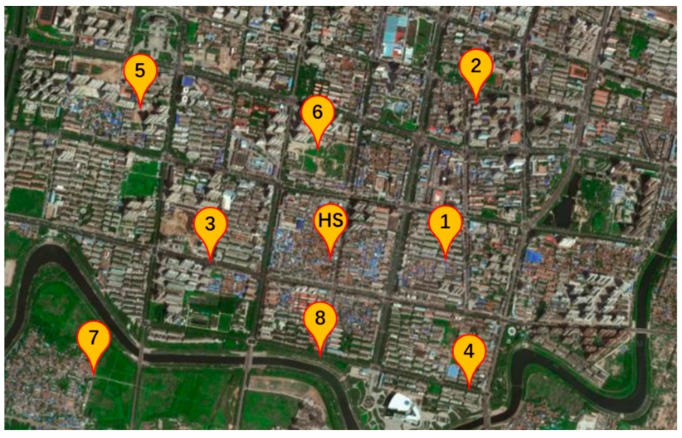
Distribution of air monitoring points. HS: HengShui station.

The experiment is designed and conducted to verify the proposed correlation analysis method. The monitoring data have been collected in an industrial park in Hebei Province, China. As shown in Figure 3, 9 monitoring points are set up in the national air monitoring grid, in which the central point (named HS station, abbreviation for HengShui station) is of higher management lever than circumjacent points. For the points, the atmospheric indexes are measured and recorded every hour, and the time range is from 1 May 2016, to 6 September 2017. The indexes consist of pollutant variables and meteorological factors. The pollutants include PM_10_, PM_2.5_, SO_2_, NO_2_, CO, O_3_, O_3_-8H (mean concentration of O_3_ in 8 h) and TVOC (Total Volatile Organic Compounds). The meteorological factors include temperature, humidity, wind direction, and wind speed.

Three parts of the experiments are designed in this paper, including multiple pollutant correlation, multiple point correlation, and multidimensional correlation. In the three experiments, parts of the monitoring indexes and points are selected as the representative application of the method.

For the correlation analysis of multiple pollutants, various variables are focused on by selecting just a monitoring point (HS station). PM_2.5_ is set as the object variable, and the relative variables include PM_10_, CO, temperature, and humidity. Then, the correlation degree between PM_2.5_ with the other four variables is calculated. In the experiment, three sections of a period (10 days) in different seasons, are analyzed, namely, the middle ten days in July 2016, December 2016, and May 2017.

For the correlation analysis of multiple points, the pollutant variable is fixed (PM_2.5_), and the points are the main analysis object. On the one hand, the relation of any two points is tested. On the other hand, HS station is mainly analyzed with four circumjacent points, including No.1 (500 m in the east), No.2 (1000 m in the northeast), No.3 (500 m in the west), and No.4 (1000 m in the southeast). The time period is the same as the previous experiment.

For the multidimensional correlation analysis, the correlation degree of different variables in various points should be analyzed. For the paper length limit, a few variables and points are selected from the previous two experiments. The selected relation to be analyzed is shown in Table 1, in which the star mark means the related matric elements will be analyzed.

Moreover, the performance of the proposed method is interpreted comparing with other methods. Firstly, the traditional static correlation analysis is set as the contrast, in which one constant degree is output based on the whole data segment. The first method is abbreviated as “static correlation”. Because the proposed method consists of the adaptive sliding window and grey relational analysis, the two parts are replaced with the classical methods respectively to form the contrast methods. Secondly, the sliding window length is fixed, referring to the traditional calculation. Then, the second contrast method is grey relational analysis with a fixed sliding window, abbreviated as “FSW-GRA”. Thirdly, another correlation method is tried to replace grey relational analysis. The classical partial correlation is selected to form the third contrast method, namely partial correlation with adaptive sliding window, abbreviated as “ASW-PC”. The proposed method in this paper is abbreviated as “ASW-GRA”. The contrast methods are conducted in some of the three experiments above.

### 4.2. Results

#### 4.2.1. Correlation of Multiple Pollutants

**Figure 4:**
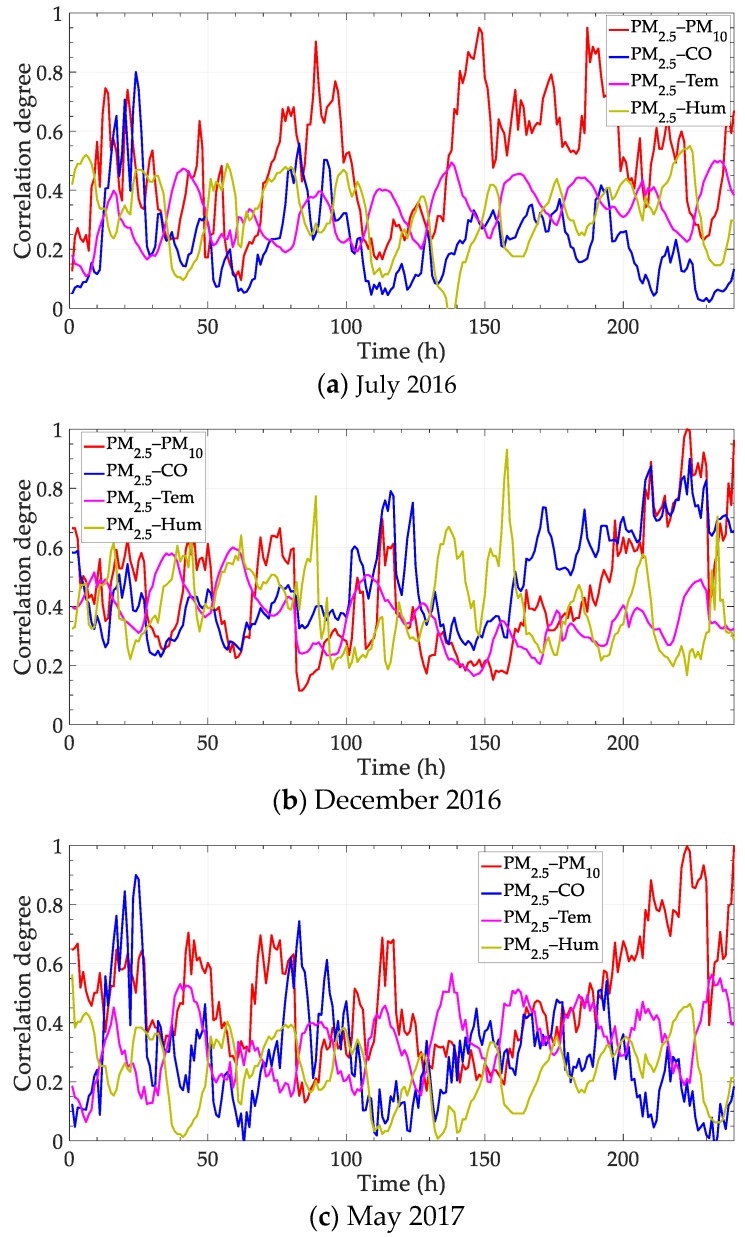
Correlation degree between PM_2.5_ and PM_10_, CO, temperature, humidity. Temperature and humidity are abbreviated as Tem and Hum, respectively.

In this part of the experiment, the correlation between different variables is analyzed in one monitoring point. Based on the experimental settings, the correlation degrees between PM_2.5_ and PM_10_, CO, temperature and humidity are calculated in three periods. The results are shown in Figure 4, in which the three subfigures are corresponding to the middle ten days of three months in different seasons.

Results in Figure 4 show the change of the influence factor on PM_2.5_ along the time. In each subfigure, the correlation degree between PM_2.5_ and the other four variables is calculated every hour, and the total number of data is 240 (10 days). The correlation degree can be ranked at each time point, and the main influence factor is not fixed at different time points. Moreover, the correlation trends are different in multiple seasons. It can be inferred from the results that a certain variable should not be determined as the only and the most important impact factor generally, but according to the time change.

**Figure 5:**
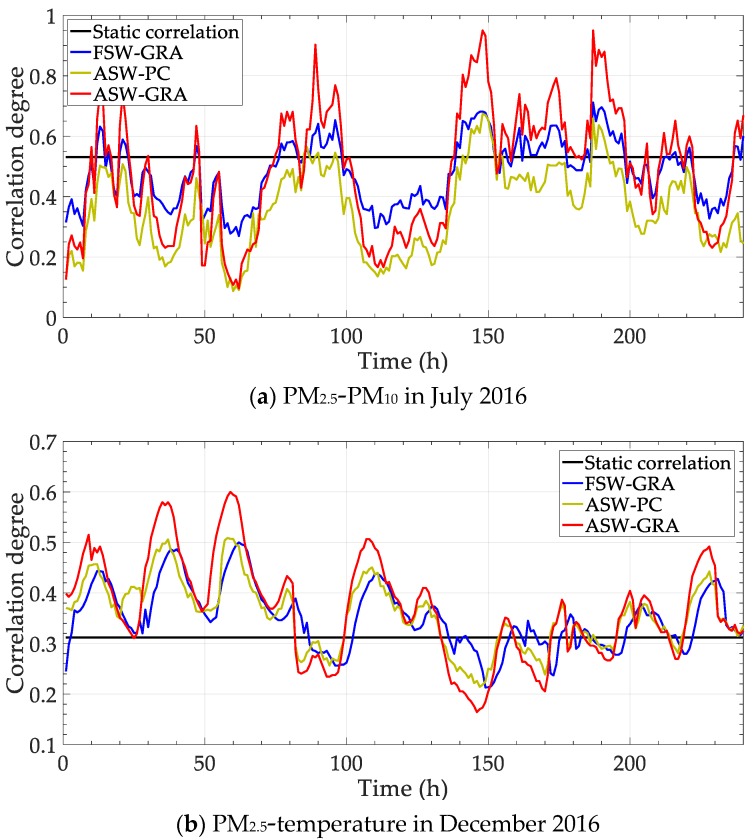
Correlation degrees by different methods.

**Figure 6:**
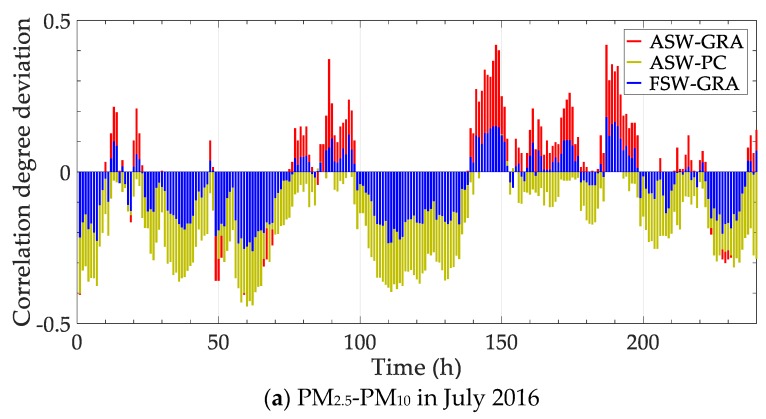

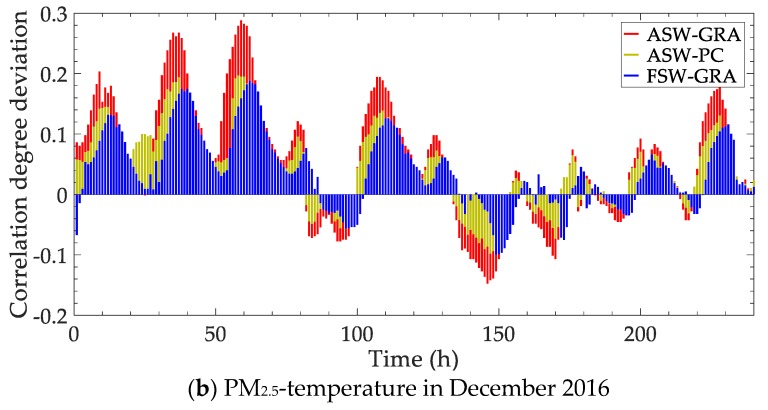
Correlation degree deviation between dynamic and static methods.

Parts of the correlation above are selected to be re-analyzed with contrast methods. Concretely, the correlations of PM_2.5_—PM_10_ in July 2016 and PM_2.5_—temperature in December 2016 are calculated with four methods, including “Static correlation”, grey relational analysis with fixed sliding window “FSW-GRA”, partial correlation with adaptive sliding window “ASW-PC” and the proposed method “ASW-GRA”. The results are shown in Figure 5. Besides, the deviation between the dynamic methods (the latter three) and the static correlation degree is calculated. The deviation is shown in Figure 6.

In Figure 5, the traditional static correlation degree cannot reflect the change over time. In fact, the main influence factor is not fixed, as shown in Figure 4. The static correlation degree may mislead the verdict of the influence factor. For dynamic performance, an obvious distinction is expected to for different time points. In this view, the fluctuation of our method (ASW-GRA) is bigger than others, which means it can represent the change more markedly. For ASW-GRA and FSW-GRA, they distinguish in the sliding window length. There is seemingly a delay for the fixed window length, which is evident in Figure 6b. For ASW-GRA and ASW-PC, they distinguish in the correlation calculation method. The deviation of ASW-GRA is larger than ASW-PC, although they perform similarly in the whole trend. The deviation shows the discrimination ability of grey relational analysis and partial correlation.

#### 4.2.2. Correlation of Multiple Points

**Figure 7:**
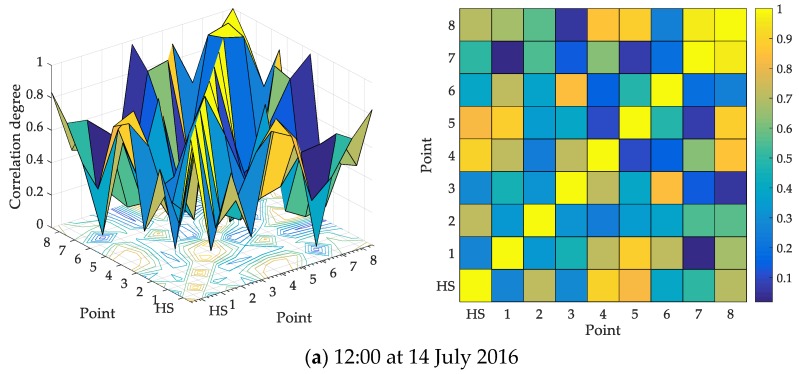

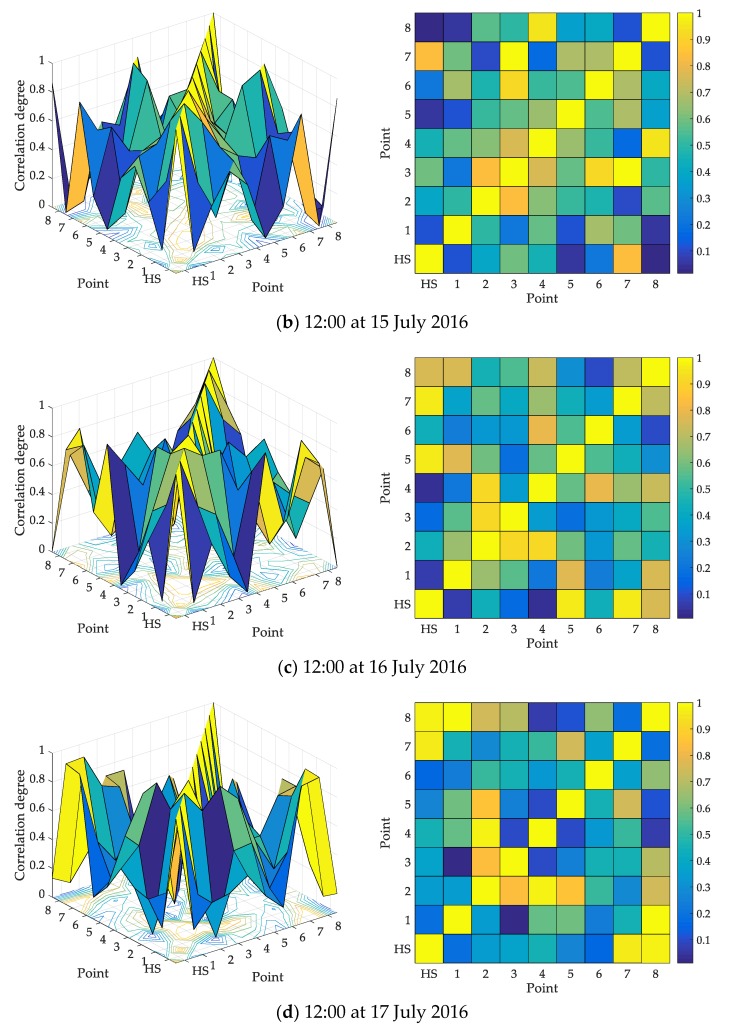
Cross-correlation degree of any two monitoring points at 4 moments.

In the experiment of multiple point correlation, the points are analyzed for the pollutant variable PM_2.5_. The correlation degree of any two points can be calculated along time, where a two-dimensional matrix will be formed at each time point. For simplicity, some results of cross-correlation degree of any two points are presented in Figure 7.

In Figure 7, the three-dimensional mesh is drawn for the cross-correlation of any two points, where the right planar graph is the x–y view of the left 3-D mesh. The color in Figure 7 represents the value of the correlation degree. For the selected time points in four days, the maximum correlation degree appears in different cross points. The yellow blocks are Point 7–8 in (a), Point 3–7 in (b), Point 5–HS, Point 7–HS in (c), and Point 1–8 in (d). It means the interaction between different positions over time, and the correlation analysis can help to ascertain the spatial influence dynamically.

**Figure 8:**
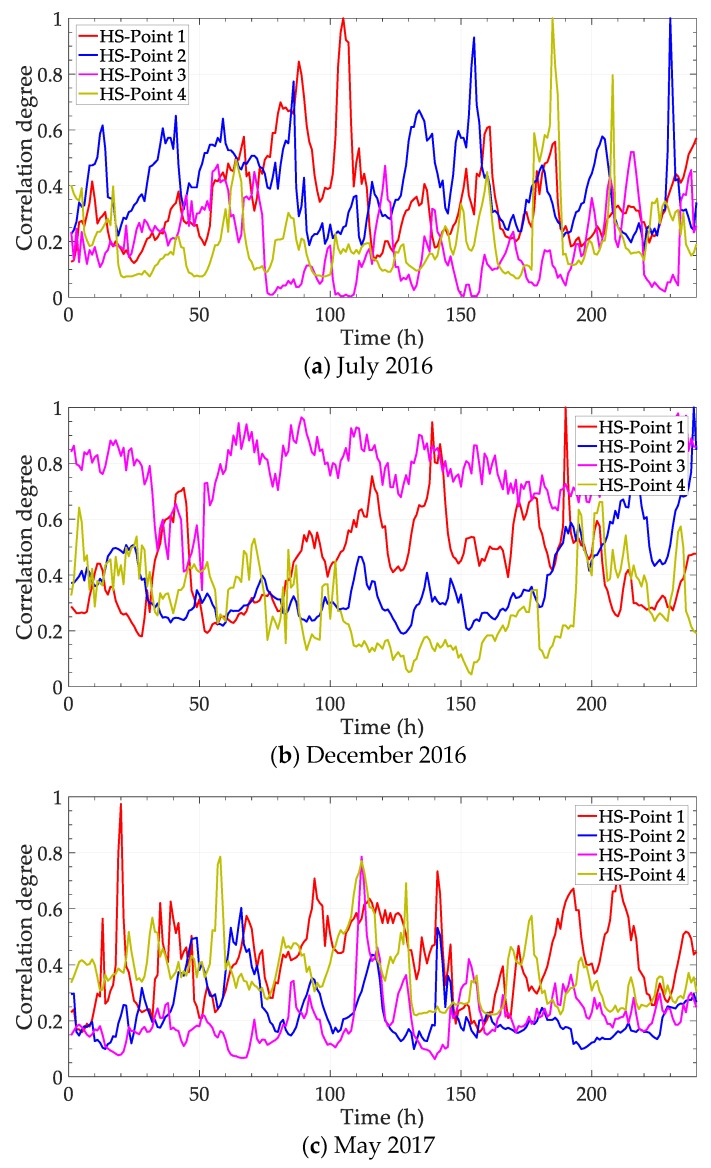
Correlation degrees between any two points.

Except for the general presentation of correlation between any two points, the object point HS station is analyzed solely with four points, and four sets of correlation degrees are obtained. The results of the three periods are shown in Figure 8.

For the correlation degree between HS station and circumjacent points, the season factor significantly reacts. There is a bright distinction in the general trend of different periods. The impact level of points can be ranked with the correlation degree. Then, it can help to deduce the direction of the pollution source. Besides, the effect of points may vary at different times. For example, in Figure 8c, Point 4 dominates from the 50th to the 60th hour, but Point 2 surpasses at the 60–70th hour.

**Figure 9:**
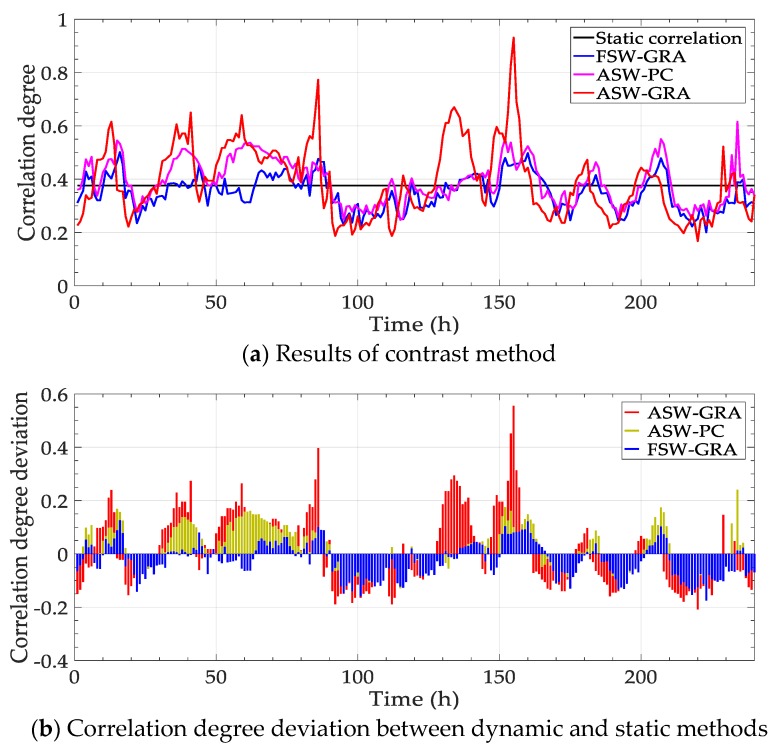
Correlation degrees between two points by contrast methods (data of July 2016).

Different dynamic contrast methods are analyzed in one period (July 2016) for HS station and No.1 point. The results of contrast methods are shown in Figure 9, of which the subfigures show the direct result and the deviation from the static correlation.

The contrast methods perform similarly with the first experiment (Figure 5 and Figure 6). The values of the deviation from ASW-GRA fluctuate more sharply than the other two. It reflects that the proposed method can distinguish the correlation degree at different time points. The dynamic property of our method can be proved again with the set of data in this part.

#### 4.2.3. Multidimensional Correlation

**Figure 10:**
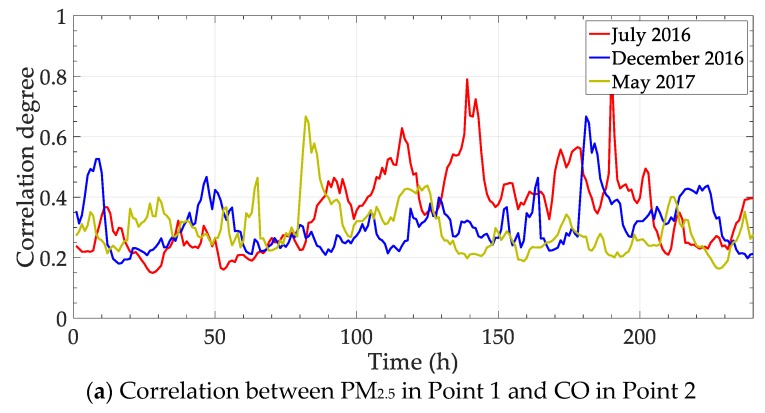

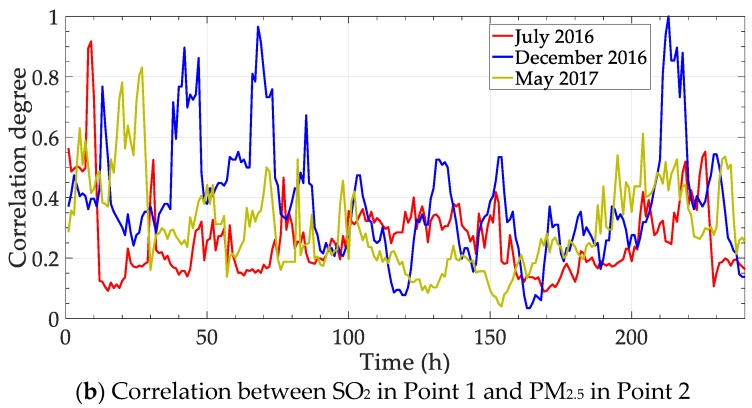
Correlation degrees of variable and point crosswise.

The previous two experiments were conducted by controlling the analysis objects, either for variables or for points. The variables and points are analyzed crosswise in this part. Following the selected elements in Table 1, the correlation degrees between PM_2.5_ in Point 1 and CO in Point 2, SO_2_ in Point 1 and PM_2.5_ in Point 2 are calculated in three periods. The results are shown in Figure 10.

**Figure 11:**
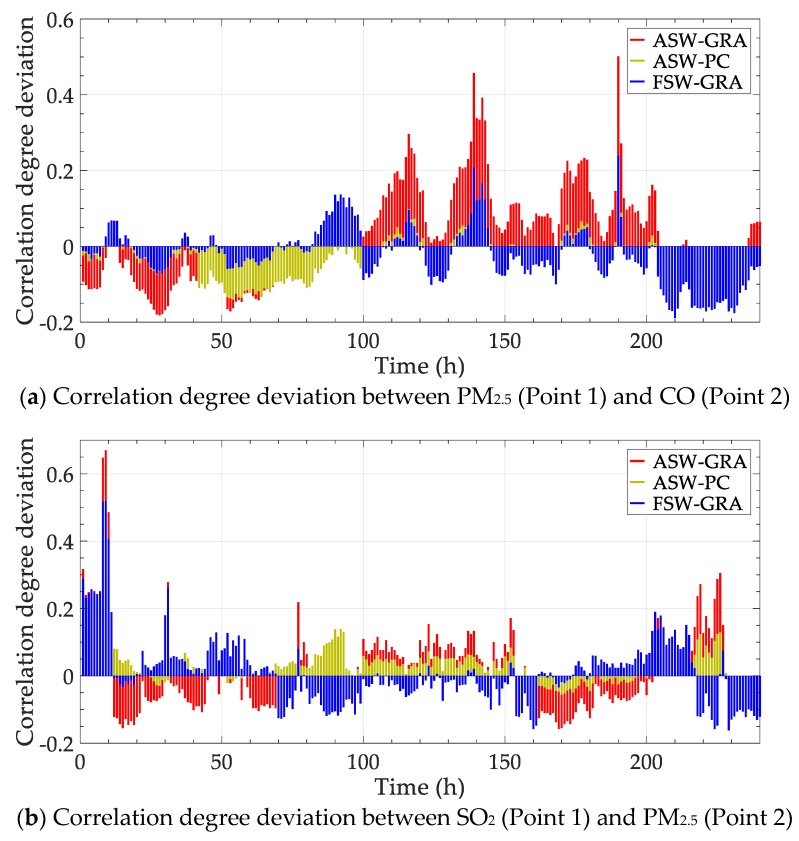
Correlation degree deviation between dynamic and static methods of data in July 2016.

The contrast methods are also conducted for the elements above (one set of data in a period is selected). The static correlation degree between PM_2.5_ (Point 1) and CO (Point 2) is 0.332, and static degree between SO_2_ (Point 1) and PM_2.5_ (Point 2) is 0.247. The deviations between the static degree and dynamic methods are shown in Figure 11.

The third experiment is conducted in the cross analysis on different pollutants in various monitoring points. The trend of correlation degree is similar to the previous experiments, including the data change and the contrast method performance. The results can help in analyzing the major influence factor from different positions. 

## 5. Discussion

Correlation analysis works weightily in atmospheric pollutant monitoring and source trace. The problem is emphatically considered; how to find out the main pollution influence factor in real-time with direct results. For a direct measurement and convenient analysis method, a dynamic correlation calculation method is proposed, which has been tested with the practical monitoring data in an industrial park of Hebei province, China.

The method can be evaluated from two aspects. On the one hand, it can reach the basic function of the traditional correlation analysis, which is reflected by that the dynamic correlation degrees distribute around the constant line in Figure 5 and Figure 9. On the other hand, the most striking feature of the proposed method is the dynamic performance, which can be found in the results of different tests. Unlike traditional statistical result, the correlation degree varies along time. It means that the impact factor on a certain pollutant variable or monitoring station is not fixed. Therefore, it is essential to obtain a real-time correlation degree to judge the main impact factor for the pollution source trace and control.

For dynamic performance, some similar methods were formed. For a quantitative comparison, the information entropy is introduced to represent the fluctuation degree. The results of the last experiment in Section 4.2.1 are analyzed with information entropy. The entropy is transformed and presented in Table 2, in which the larger the value, the larger the fluctuation degree. It reflects that the proposed method distinguishes the correlation degrees of each time point. The apparent change helps to find out the most relevant influence factors over time. The feature of the results is the specific performance of the dynamic property in the proposed method.

For the paper length limitation, only some variables and points are selected and presented. In fact, the proposed method can be applied to the correlation analysis of any two factors in the same type. For example, PM_2.5_ is analyzed with four variables in Section 4.2.1, but any two of the five variables can be calculated following the proposed algorithm. In general, the proposed method is essential for the correlation between variables, which is not limited by the examples in the experiment. In fact, the method has been encapsulated as a program in the information management system of an industrial park in Hebei Province [36]. In the information system, multiple variables can be analyzed following the proposed method, from the view of pollutants and positions. The function of dynamic correlation analysis in the information system has helped administrators to trace the pollution source. Besides, the proposed method can provide the decision-making support with other system functions of the real-time monitoring and trend prediction [37,38].

For the method to calculate the monitoring data iteratively in real-time, there is a requirement for the computing resource with high performance. In the future, the improvement can be carried out to reduce the calculated amount. Then, the method can be applied widely in small-scale systems and low-performance terminals. Besides, the method analyzes the correlation degree in discrete points. When there is a need for the continuous distribution of the atmosphere, other gas diffusion methods should be explored to integrate with the method.

## 6. Conclusions

For the atmospheric management issue of pollutant interaction and source tracing, a dynamic correlation analysis method is proposed. It is designed with a convenient process and direct result measurement. The proposed method realizes the relation extraction for pollutant variables in real-time, as well as the space factors, which have been tested with the practical monitoring data. The method is an effective support for air quality management in the modern information era. It provides the reference framework for the emerging pollutant and source for air quality. The correlation result can help pollution control and sustainable planning. In future work, the method can be applied in other analyses of new variables, such as particulate matter, nitrogen oxides, traffic emission, and consumer products. Besides, the method can be explored with the continuous analysis models, which can output the fine-grained results of the atmosphere diffusion. The improved correlation analysis method will support pollution management with information mining.

## Figures and Tables

**Table 1:** Variable and point selected as the analysis object of multidimensional correlation.

		Point No.1		Point No.2	
		PM_2.5_	SO_2_	PM_2.5_	CO
Point No.1	PM_2.5_				★
	SO_2_			★	
Point No.2	PM_2.5_		★		
	CO	★			

★: The related matric elements will be analyzed.

**Table 2:** Information entropy of contrast methods in experiment 1 (Section 4.2.1).

Period	FSW-GRA	ASW-PC	ASW-GRA
PM_2.5_-PM_10_ in July 2016	0.476	0.598	0.869
PM_2.5_-temperature in December 2016	0.511	0.547	0.763

FSW-GRA: grey relational analysis with a fixed sliding window; ASW-PC: partial correlation with adaptive sliding window; ASW-GRA: the proposed method in this paper, namely gray relation analysis with adaptive sliding window.

## References

[1] Hopke P.K., Ito K., Mar T., Christensen W.F., Eatough D.J., Henry R.C., Kim E., Laden F., Lall R., Larson T.V. (2006). PM source apportionment and health effects: 1. Intercomparison of source apportionment results. J. Expo. Sci. Environ. Epidemiol..

[2] Shumake K.L., Sacks J.D., Lee J.S., Johns D.O. (2013). Susceptibility of older adults to health effects induced by ambient air pollutants regulated by the European Union and the United States. Aging Clin. Exp. Res..

[3] Kim E., Hopke P.K., Pinto J.P., Wilson W.E. (2005). Spatial variability of fine particle mass, components, and source contributions during the regional air pollution study in St. Louis. Environ. Sci. Technol..

[4] Hwang I., Hopke P.K., Pinto J.P. (2008). Source apportionment and spatial distributions of coarse particles during the regional air pollution study. Environ. Sci. Technol..

[5] Shang X., Li Y., Pan Y., Liu R.F., Lai Y.P. (2013). Modification and application of gaussian plume model for an industrial transfer park. Adv. Mater. Res..

[6] Cao X., Roy G., Hurley W.J., Andrews W.S. (2011). Dispersion coefficients for Gaussian puff models. Bound. Layer Meteorol..

[7] Poulsen T.G., Christophersen M., Moldrup P., Kjeldsen P. (2003). Relating landfill gas emissions to atmospheric pressure using numerical modelling and state-space analysis. Waste Manag. Res. J. Int. Solid Wastes Public Clean. Assoc. Iswa.

[8] Farrell J.A., Pang S., Li W. (2003). Plume mapping via hidden Markov methods. IEEE Trans. Syst. Manand Cybern. Part B.

[9] Wikle C.K., Zammit-Mangion A., Cressie N. (2019). Spatio-Temporal Statistics with R.

[10] Hefley T.J., Hooten M.B., Hanks E.M., Russell R.E., Walsh D.P. (2017). Dynamic spatio-temporal models for spatial data. Spat. Stat..

[11] Cressie N., Wikle C.K. (2011). Statistics for Spatio-Temporal Data.

[12] Mateu J., Giraldo R. (2019). Geostatistical Functional Data Analysis: Theory and Methods.

[13] Ramsay J.O., Silverman B.W. (2007). Applied Functional Data Analysis: Methods and Case Studies.

[14] Baba K., Shibata R., Sibuya M. (2004). Partial correlation and conditional correlation as measures of conditional independence. Aust. N. Z. J. Stat..

[15] Wold S., Esbensen K., Geladi P. (1987). Principal Component analysis. Chemom. Intell. Lab. Syst..

[16] Kuo Y., Yang T., Huang G. (2008). The use of grey relational analysis in solving multiple attribute decision-making problems. Comput. Ind. Eng..

[17] Brusca S., Famoso F., Lanzafame R., Mauro S., Garrano A.M.C., Monforte P. (2016). Theoretical and experimental study of gaussian plume model in small scale system. Energy Procedia.

[18] Hosseini B., Stockie J.M. (2016). Bayesian estimation of airborne fugitive emissions using a Gaussian plume model. Atmos. Environ..

[19] Guo D., Yu J., Ban M. (2018). Security-constrained unit commitment considering differentiated regional air pollutant intensity. Sustainability.

[20] Ramsay J., Hooker G. (2017). Dynamic Data Analysis—Springer Series in Statistics.

[21] Bohorquez M., Giraldo R., Mateu J. (2016). Optimal sampling for spatial prediction of functional data. Stat. Methods Appl..

[22] Giraldo R., Delicado P., Mateu J. (2011). Ordinary kriging for function-valued spatial data. Environ. Ecol. Stat..

[23] Li X., Qiu T., Chen G., Zhong L.X., Wu X.R. (2016). Market impact and structure dynamics of the Chinese stock market based on partial correlation analysis. Phys. A Stat. Mech. Its Appl..

[24] Rahmani M., Atia G. (2016). Coherence pursuit: Fast, simple, and robust principal component analysis. IEEE Trans. Signal Process..

[25] Tang J., Zhu H., Liu Z., Jia F., Zheng X.X. (2019). Urban sustainability evaluation under the modified TOPSIS based on grey relational analysis. Int. J. Environ. Res. Public Health.

[26] Porth I., White R., Jaquish B., Ritland K. (2018). Partial correlation analysis of transcriptomes helps detangle the growth and defense network in spruce. New Phytol..

[27] Olszewski A., Broniowski W. (2017). Partial correlation analysis method in ultrarelativistic heavy-ion collisions. Phys. Rev. C.

[28] Calce S.E., Kurki H.K., Weston D.A., Gould L. (2017). Principal Component analysis in the evaluation of osteoarthritis. Am. J. Phys. Anthropol..

[29] Lionnie R., Alaydrus M. Biometric Identification System Based on Principal Component Analysis. Proceedings of the 2016 12th International Conference on Mathematics, Statistics, and Their Applications (ICMSA).

[30] Cai L., Thornhill N.F., Kuenzel S., Pal B.C. (2018). Wide-area monitoring of power systems using principal component analysis and k-nearest neighbor analysis. IEEE Trans. Power Syst..

[31] Fu B., Gao X., Wu L. (2018). Grey relational analysis for the AQI of Beijing, Tianjin, and Shijiazhuang and related countermeasures. Grey Syst. Theory Appl..

[32] Cao X., Deng H., Lan W. (2015). Use of the grey relational analysis method to determine the important environmental factors that affect the atmospheric corrosion of Q235 carbon steel. Anti-Corros. Methods Mater..

[33] Hashemi S.H., Karimi A., Tavana M. (2015). An integrated green supplier selection approach with analytic network process and improved grey relational analysis. Int. J. Prod. Econ..

[34] Malekpoor H., Chalvatzis K., Mishra N., Mehlawat M.K., Zafirakis D., Song M. (2018). Integrated grey relational analysis and multi objective grey linear programming for sustainable electricity generation planning. Ann. Oper. Res..

[35] Wang H., Guo L., Dou Z., Lin Y. (2018). A new method of cognitive signal recognition based on hybrid information entropy and DS evidence theory. Mob. Netw. Appl..

[36] Bai Y., Wang X., Sun Q., Jin X.B., Wang X.K., Su T.L., Kong J.L. (2019). Spatio-Temporal prediction for the monitoring-blind area of industrial atmosphere based on the fusion network. Int. J. Environ. Res. Public Health.

[37] Jin X., Yang N., Wang X., Bai Y., Su T., Kong J. (2019). Integrated predictor based on decomposition mechanism for PM2.5 long-term prediction. Appl. Sci..

[38] Bai Y., Jin X., Wang X., Su T., Kong J., Lu Y. (2019). Compound autoregressive network for prediction of multivariate time series. Complexity.

